# Testing ZigBee Motes for Monitoring Refrigerated Vegetable Transportation under Real Conditions

**DOI:** 10.3390/s100504968

**Published:** 2010-05-18

**Authors:** Luis Ruiz-Garcia, Pilar Barreiro, Jose Ignacio Robla, Loredana Lunadei

**Affiliations:** 1 Laboratorio de Propiedades Físicas y Tecnologías Avanzadas en Agroalimentación, Universidad Politécnica de Madrid, ETSI Agrónomos, Edificio Motores, Avda. Complutense s/n 28040 Madrid, Spain; E-Mail: pilar.barreiro@upm.es; 2 Centro Nacional de Investigaciones Metalúrgicas (CENIM-CSIC), Avda. Gregorio del Amo, 8, 28040, Madrid, Spain; E-Mail: jrobla@cenim.csic.es; 3 Dipartimento di Ingegneria Agraria, Università degli Studi di Milano, Via Celoria, 2-20133 Milano, Italy; E-Mail: loredana.lunadei@gmail.com

**Keywords:** ZigBee, wireless sensing, cold chain, logistics, food

## Abstract

Quality control and monitoring of perishable goods during transportation and delivery services is an increasing concern for producers, suppliers, transport decision makers and consumers. The major challenge is to ensure a continuous ‘cold chain’ from producer to consumer in order to guaranty prime condition of goods. In this framework, the suitability of ZigBee protocol for monitoring refrigerated transportation has been proposed by several authors. However, up to date there was not any experimental work performed under real conditions. Thus, the main objective of our experiment was to test wireless sensor motes based in the ZigBee/IEEE 802.15.4 protocol during a real shipment. The experiment was conducted in a refrigerated truck traveling through two countries (Spain and France) which means a journey of 1,051 kilometers. The paper illustrates the great potential of this type of motes, providing information about several parameters such as temperature, relative humidity, door openings and truck stops. Psychrometric charts have also been developed for improving the knowledge about water loss and condensation on the product during shipments.

## 1. Introduction

Perishable food products such as vegetables, fruit, meat or fish require refrigerated transportation. For all these products, Temperature (T) is the most important factor for extending shelf life, being essential to ensure that temperatures along the cold chain are adequate. However, local temperature deviations can be present in almost any transport situation. Reports from the literature indicate gradients of 5 °C or more, when deviations of only a few degrees can lead to spoiled goods and thousands of Euros in damages [1–4]. A recent study shows that refrigerated shipments rise above the optimum temperature in 30% of trips from the supplier to the distribution centre, and in 15% of trips from the distribution centre to the stores [5]. Roy *et al.* analyzed the supply of fresh tomato in Japan and quantified product losses of 5% during transportation and distribution [6]. Thermal variations during transoceanic shipments have also been studied. The results showed that there was a significant temperature variability both spatially across the width of the container as well as temporally along the trip, and that it was out of the specification more than 30% of the time. In those experiments monitoring was achieved by means of the installation of hundreds of wired sensors in a single container, which makes this system architecture commercially unfeasible [1,7].

Transport is often done by refrigerated road vehicles and containers equipped with embedded cooling systems. In such environments, temperatures rise very quickly if a reefer unit fails [8]. Commercial systems are presently available for monitoring containers and trucks, but they do not give complete information about the cargo, because they typically measure only temperature and at just one point [9].

Apart from temperature, water loss is one of the main causes of deterioration that reduces the marketability of perishable food products. Transpiration is the loss of moisture from living tissues. Most weight loss of stored fruit is caused by this process. Relative humidity (RH), T of the product, T of the surrounding atmosphere, and air velocity all affect the amount of water lost in food commodities. Free water or condensation is also a problem as it encourages microbial infection and growth, and it can also reduce the strength of packaging materials [10].

Parties involved need better quality assurance methods to satisfy customer demands and to create a competitive point of difference. Successful transport in food logistics calls for automated and efficient monitoring and control of shipments. The challenge is to ensure a continuous ‘cold chain’ from producer to consumer in order to guaranty prime condition of goods [9].

The use of wireless sensors in refrigerated vehicles was proposed by Qingshan *et al*. as a new way of monitoring [11]. Specialized WSN (Wireless Sensor Network) monitoring devices promise to revolutionize the shipping and handling of a wide range of perishable products giving suppliers and distributors continuous and accurate readings throughout the distribution process. In this framework, ZigBee was developed as a very promising WSN protocol due to its low energy consumption and advanced network capabilities. Its potential for monitoring the cold chain has been addressed by several authors but without real experimentation, only theoretical approaches [11–13]. For this reason, in our work real experimentation with the aim of exploring the limits of this technology was a priority.

The main objective of this project is to explore the potential of wireless ZigBee/IEEE 802.15.4 motes for their application in commercial refrigerated shipments by road. A secondary objective was to improve the knowledge about the conditions that affect the perishable food products during transportation, through the study of relevant parameters like temperature, relative humidity, light, shocking and psychrometric properties.

## 2. Materials and Methods

### 2.1. ZigBee Motes

Four ZigBee/IEEE 802.15.4 motes (transmitters) and one base station (receiver) were used. All of them were manufactured by Crossbow. The motes consist of a microcontroller board (Micaz) together with an independent transducer board (MTS400) attached by means of a 52 pin connector. The Micaz mote hosts an Atmel ATMEGA103/128L CPU running the Tiny Operating System (TinyOS) that enables it to execute programs developed using the nesC language. The Micaz has a radio device Chipcon CC2420 2.4 GHz 250 Kbps IEEE 802.15.4. Power is supplied by two AA lithium batteries.

The transducer board hosts a variety of sensors: T and RH (Sensirion SHT11), T and barometric pressure (Intersema MS5534B), light intensity (TAOS TSL2550D) and a two-axis accelerometer (ADXL202JE). A laptop computer is used as the receiver, and communicates with the nodes through a Micaz mounted on the MIB520 ZigBee/USB gateway board.

Each Sensirion SHT11 is individually calibrated in a precision humidity chamber. The calibration coefficients are used internally during measurements to calibrate the signals from the sensors. The accuracies for T and RH are ±0.5 °C (at 25 °C) and ±3.5% respectively.

The Intersema MS5534B is a SMD-hybrid device that includes a piezoresistive pressure sensor and an ADC-Interface IC. It provides a 16 bit data word from a pressure and T (−40 to +125°C) dependent voltage. Additionally the module contains six readable coefficients for a highly accurate software calibration of the sensor.

The TSL2550 is a digital-output light sensor with a two-wire, SMBus serial interface. It combines two photodiodes and an analog-to digital converter (ADC) on a single CMOS integrated circuit to provide light measurements over a 12-bit dynamic range. The ADXL202E measures accelerations with a full-scale range of ±2 g. The ADXL202E can measure both dynamic acceleration (e.g., vibration) and static acceleration (e.g., gravity).

### 2.2. Experimental Set Up

The experiment was conducted in a refrigerated truck traveling during 23 h 41 m 21 s from Murcia (Spain) to Avignon (France), a distance of 1,051 km. The truck transported approx.14,000 kg of lettuce var. *Little Gem* in 28 pallets of 1,000 × 1,200 mm. The lettuce was packed in cardboard boxes with openings for air circulation.

The length of the semi-trailer was 15 m with a Carrier Vector 1800 refrigeration unit mounted to the front of the semi-trailer. For this shipment the set point was 0 °C.

**Figure 1.**
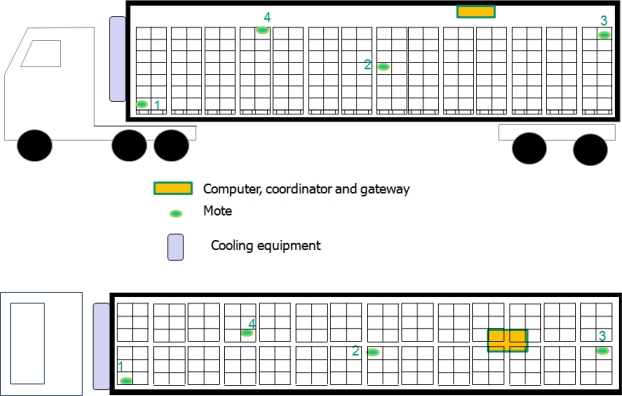
Experimental set-up. Side view and top view.

The truck was outfitted with the wireless system, covering different heights and lengths from the cooling equipment, which was at the front of the semi-trailer. Four motes were mounted with the cargo (see Figure 1): mote 1 was at the bottom of the pallets in the front side of the semi-trailer, mote 2 was in the middle of the semi-trailer, mote 3 was in the rear at the top of the pallet, and mote 4 was located as shown in Figure 1, about a third of the distance between the front and the rear of the trailer. Motes 1, 2 and 3 were inside the boxes beside the lettuce. The program installed in the motes collects data from all the sensors at a fixed sample rate (7.2 s), with each transmission referred to as a “*packet*”. The RF power in the Micaz can be set from −24 dBm to 0 dBm. During the experiment, the RF power was set to the maximum, 0dBm (1mW approximately).

### 2.3. Data Analysis

A specialized MATLAB program has been developed for assessing the percentage of lost packets (%) in transmission, by means of computing the number of multiple sending failures for a given sample rate (*SR*). A multiple failure of *m* messages occurs whenever the elapsed time between two messages lies between 1.5 × *m* × *SR* and 2.5 × *m* × *SR*. For example, with a sample rate of 11 s, a single failure (m = 1) occurs whenever the time period between consecutives packets is longer than 16.5 s (1.5 × 1 × 11) and shorter than 27.5 s (2.5 × 1 × 11). The total number of lost packets is computed based on the frequency of each failure type. Accordingly, the total percentage of lost packets is calculated as the ratio between the total number of lost packets and the number of sent packets.

The standard error (*SE*) associated to the ratio of lost packets is computed based on a binomial distribution as expressed in Equation 1, where *n* is the total number of packets sent, and *p* is the ratio of lost packets in the experiment:

(1) $$\mathrm{SE}=\sqrt{\frac{p(1-p)}{n}}$$

### 2.4. Analysis of Variance

Factorial Analysis of Variance (ANOVA) was performed in order to evaluate the effect of the type of sensor in the registered measurements, including T (by means of Sensirion and Intersema), RH, barometric pressure, light intensity and acceleration module. ANOVA allows partitioning of the observed variance into components due to different explanatory variables. The STATISTICA software (StatSoft, Inc.) was used for this purpose [14]. The Fishers’s F ratio compares the variance within sample groups (“inherent variance”) with the variance between groups (factors). We use this statistic for knowing which factor has more influence in the variability of the measurements.

### 2.5. Psychrometric Data

Psychrometry studies the thermodynamic properties of moist air and the use of these properties to analyze conditions and processes involving moist air. Psychrometric charts show a graphical representation of the relationship between T, RH and water vapor pressure in moist air [15]. They can be used for the detection of water loss and condensation over the product [16].

In our study, the ASAE standard D271.2 was used for computing the psychrometric properties of air [15]. Equations 2–5 and Table 1 enable the calculation of all psychrometric data of air whenever two independent psychrometric properties of an air-water vapour mixture are known in addition to the atmospheric pressure:

(2) $$\begin{array}{l} \mathrm{Ps}=e^{31.9602-\frac{6270.3605}{T}-0.46057*\text{ln}\;T}\\ \;\;\;\;\;\;\;-255.38\;K\leq T\leq 273.16\;K \end{array}$$

(3) $$\begin{array}{l} \mathrm{Ps}=R*e^{\frac{A+B*T+C*T^{2}+D*T^{3}+E*T^{4}}{F*T-G*T^{2}}}\\ \;\;\;\;\;\;\;\;273.16\;K\leq T\leq 533.16K \end{array}$$

(4) $$\mathrm{Pv}=\mathrm{Ps}\frac{\mathrm{RH}}{100}$$

(5) $$H=\frac{0.6219*\;\mathrm{Pv}}{\mathrm{Patm}-\mathrm{Pv}}$$

where *Ps* stands for saturation vapor pressure (Pa), *T* is the temperature (K), *Pv* is the vapor pressure (Pa), *H* the absolute humidity (g/kg dry air), *Patm* is atmospheric pressure (Pa) and A, B, C, D, E, F, G and R are a series of coefficients used to compute *Ps*, according to Equation 3 (see Table 1).

## 3. Results and Discussion

### 3.1. Reliability of Transmission

Signal propagation through the lettuce lead to absorption of radio signals, resulting in great attenuations in RF signal strength and link quality at the receiver. During the experiment, only motes 3 and 4 were able to transmit to the coordinator. No signals were received from mote number 1, at the bottom of the first pallet, and number 2, in the middle of the pallet. Mote 3 was closer to the coordinator than mote 4, but mote 3 was surrounded by lettuce which blocks the RF signal. However between mote 4 and the coordinator there was free space for transmission. Thus, the maximum ratio of lost packets found was 100% for two of the motes and the minimum 4.5% ± 0.1%, for mote 4.

Similar ratios were reported by several authors who performed experiments with WSN under real conditions, like for example in monitoring vineyards [17]. Also, Baggio [18] and Haneveld [19], after one year of experimentation in a potato field using motes operating at the band of 868/916MHz, reported that 98% of data packets were lost. However, during the second year the total amount of data gathered was 51%, which represents a clear improvement. Ipema *et al*. monitored cows with Crossbow motes, and found that the base station directly received less than 50% of temperature measurements stored in the mote buffer [20]. Nadimi *et al*., who also monitored cows with this type of motes, showed packet loss rates of about 25% for wireless sensor data from cows in a pasture even the distance to the receiver (gateway) was less than 12.5 m away [21].

Radio propagation can be influenced by two main factors: the properties of propagation media and the heterogeneous properties of devices [22]. In a commercial shipment, if the motes are embedded within the cargo, a significant portion of the Fresnel zone is obstructed. This is a big challenge in our application. Changing the motes’ location, for example the one at the bottom of the pallets (mote 1, at the front of the semitrailer) or the one in the middle of the compartment (mote 2), might have yielded in better data reception rates but would have resulted in a loss of spatial information near the floor or at mid-height. The sensors should be as close as possible to the products transported; otherwise the measurements would not give precise information. Thus, one solution, if the same motes are to be used, could be to include intermediates motes that allow peer to peer communication to the base station. Another solution could be to use lower frequencies; however this is not possible using ZigBee, because the only radio frequency band available for ZigBee worldwide is the 2.4 GHz one. The other ISM (Industrial, Scientific and Medical) bands (868 MHz and 915 MHz) differ from USA to Europe [23,24]. Other options include developing motes with more RF power that can achieve longer radio ranges. The transmission could also be improved by optimizing antenna orientation, shape and configuration [25]. The standard antenna mounted in the Micaz is a 3 cm long 1/2 wavelength dipole antenna. The communications could be enhanced using ceramic collinear antennas [26] or with use of a simple reflecting screen to supplement a primary antenna, which can provide a 9dB improvement [27]. Link asymmetry and an irregular radio range can be caused by the antenna position. In a real environment, the pattern of radio transmitted at the antenna is neither a circular nor a spherical shape. Radio irregularity affects the motes performance and degrades their ability to maintain connection to other nodes in the network. However, in our experiment Micaz motes were deployed in its best position according to a recent study [28]. Another issue is the received signal strength indicator (RSSI), it should be recorded in further experiments in order to detect network problems and estimate the radio link quality. RSSI is a way for the radio to report the strength of the radio signal that it is receiving from the transmitting unit.

Sample rates configured in the motes were very short in order to get the maximum amount of data about the ambient conditions. In practice, a reduction in the sampling frequency of recording and transmission should be configured in order to extend battery life. According to Thiemjarus and Yang this also provides opportunities for data reduction at the mote level. It is expected that future wireless sensor motes will have on-board features to analyze recorded data and detect certain deviations. The level of a deviation determines whether the recording or transmitting frequency should be adapted [29].

One important feature in the motes came from the miniaturized sensors mounted on the motes that allow, in a small space (2.5 × 5 × 5 cm), to provide data not just about temperature, but also RH, acceleration and light, according to the proposal of Wang and Li [30]. Those variables were also measured and analyzed.

### 3.2. Transport Conditions

For the analysis of T conditions, the average value of the two sensors mounted in each mote is considered. The set-point of the transport trailer’s cooling system was 0 °C, but the average temperature registered during the shipment was 5.33 °C, with a maximum of 8.52 °C and a minimum of −3.0 °C. On average, 98% of the time the temperature was outside of the industry recommended range (set-point ± 0.5 °C).

**Figure 2.**
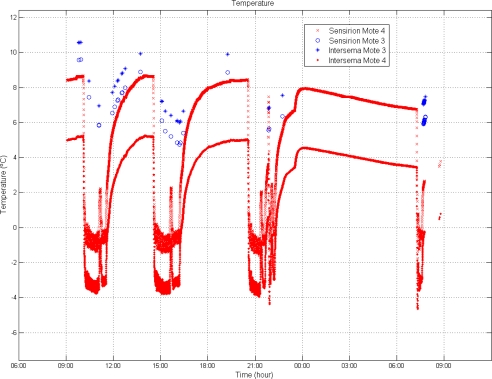
T comparison between motes and sensors.

Figure 2 shows the temperature fluctuations registered during the shipment, where four different markers are used corresponding to two T sensors per mote. There are large differences between the temperatures recorded with each sensor on the same mote even thought individual calibration curves were used. The SHT11 measures consistently higher temperatures than the Intersema. This behaviour could be due to the closer location of the SHT11 to the microcontroller, causing sensor self-heating effects.

In other studies, like for example Tanner and Amos, it was observed that the cargo was within the industry recommended T interval for approximately 58% of the shipment duration [1]. Rodriguez-Bermejo *et al.* compared two different cooling modes in a 20′ reefer container. For modulated cooling the percentage of time within the recommendation ranged between 44% and 52% of the shipment duration, whereas for off/on control cooling it ranged between 9.6% and 0%. In those experiments, lower percentages of time within industry recommended intervals are found for high T set points [3].

The analysis of variance of the T data (Table 2) shows that the variability in temperature depended both in the type of sensor and on the mote used. The interaction between these two factors also has an impact on the T measurements. The critical value of *F* at 95% probability level is much lower than the observed values of *F*, which means that the null hypothesis is false. The mote is the factor that has most influence on the variability of the measurements (highest Fishers’s F); this fact seems to be due to the location of the node. Mote 4 is closer to the cooling equipment which results in lower temperature measurements.

The node is a very significant factor in the measurements registered. In the case of RH, pressure, light and acceleration, the node location has great influence in data variability (Table 3). However, node location has more impact on the measured RH than on the other variables.

**Figure 3.**
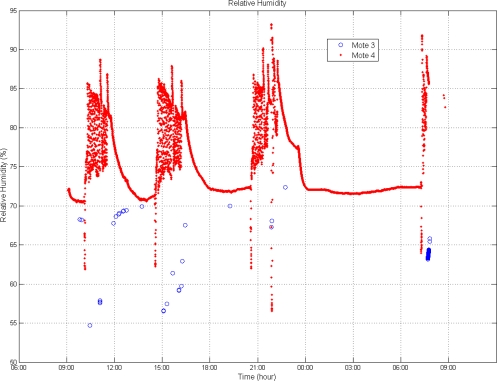
RH comparison between motes.

Inside the semi-trailer RH ranged from 55 to 95% (see Figure 3). The optimal RH for lettuce is 95%. Humidity was always higher at mote 4 (at the top middle of the semi-trailer; average RH 74.9%) than at mote 3 (located at the rear; average RH 62.1%).

**Figure 4.**
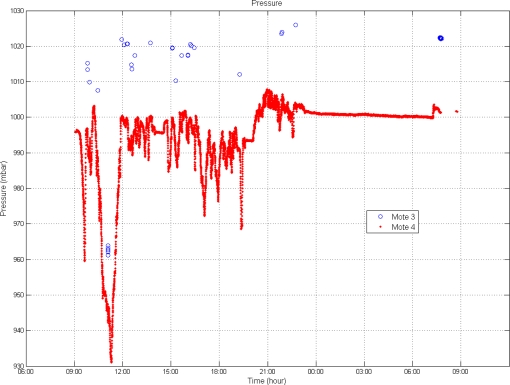
Air pressure comparison between motes.

As it was expected, the pressure oscillations at mote 3 are shorter than at mote 4 (Figure 4) located directly in contact with the air channels. This situation also corresponds with shorter temperature variations (Figure 4). The temperature increase at the end of the transportation (Figure 2) does not correspond with an increase in pressure (Figure 4) because this situation was during unloading, and the cargo was out of the truck.

When the air speed is higher, which means the reefer equipment is blowing, the pressure is lower. For this reason the pressure registered by the mote 4, which is closer to the reefer equipment, is lower than that registered by mote 3. Pressure drops corresponds to changes in the intensity of cooling.

**Figure 5.**
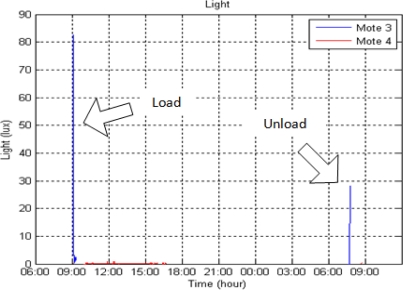
Light detection during the shipment.

Figure 5 shows intensity of light registered with the ambient light sensor. This sensor provides information about events such as loading and unloading, both characterized by door openings. In this way, an unauthorized opening of the doors could be detected.

In the proposal for a smart container by Craddock and Stansfield, several sensors for security issues were included, such as door opening sensors, volume intrusion alarms, microwave radar, ultrawide band radar, and sound or explosives sensors. But this topic falls outside the scope of this paper and should be treated as an independent system that could be installed not just in refrigerated transports, but in special kinds of transports, including very sensitive ones [31].

**Figure 6.**
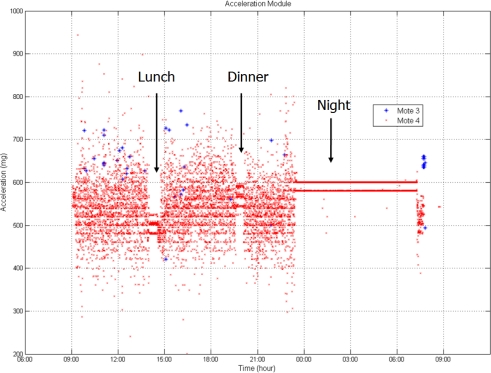
Acceleration module for the two motes.

The data registered with the accelerometers (Figure 6) show clearly when the truck was stopped and when the truck was moving. There were two short stops for lunch and dinner, and a long one during the night.

Unfortunately, there is no published data on vibrations recorded during lettuce transportation that could be compared with the data shown in Figure 6. Some studies that analyzed the vibrations transmitted from a typical truck during transportation of fruit, founding the highest acceleration values at the rear position of the semi-trailer and the lowest at the front [32]. That situation is similar with the data presented in this paper, which show higher acceleration values in the mote 3 (rear) than in mote 4 (middle). But this behaviour cannot be confirmed due to the lack of data from mote 1 (front).

### 3.3. Psychrometry

**Figure 7.**
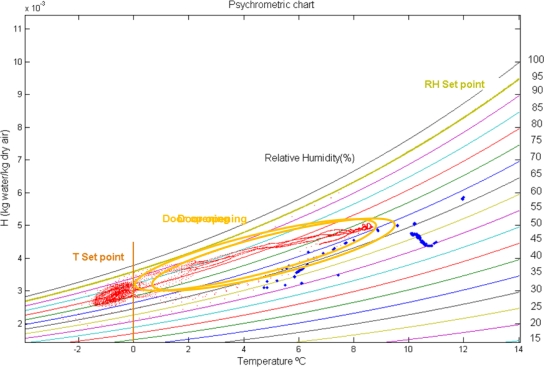
Psychrometric chart (color lines indicate relative humidity; red dots represent mote 4 and blue dots mote 3).

The absolute humidity of the air in the trailer was calculated, based on the ASAE standard D271.2 [15], using the data recorded during shipment. Psychrometric charts which illustrate the evolution of air absolute humidity (H, kg of water/kg of dry air) related to the T (°C) inside the semi-trailer for the two motes (3 and 4) are included in Figure 7. Door opening, at the rear of the truck, created an increment in T (°C) and H (kg of water/ kg of dry air), which then returned to normal again once the door is closed. During the rest of the time, it is also possible to detect the interaction between air properties and the product; with the cycles of cooling, variations in the absolute humidity can be estimated: condensation over the products (as loss of absolute humidity), or water evaporation (as an increase in absolute air humidity).

## 4. Conclusions

A commercial transport event between Murcia (Spain) and Avignon (France), was monitored throughout its whole duration. To our best knowledge, this is the first research work performed in cold chain transports using WSN technology. This study proves the potential of ZigBee/IEEE 802.15.4 motes for this application, providing a set of data never available before, including T, RH, door openings, truck stops and psychrometry. The information provided was valuable and useful for the three companies involved: the Spanish wholesaler, carrier and the French distributor. However, the system architecture were not able to fully monitor ambient conditions in a shipment of lettuce *var. Little Gem,* so investigation of the spatial variability of data was not completed achieved.

During the shipment, there were T deviations compared to the optimal conditions for the product transported. The set-point was 0 °C, but the average temperature registered during the shipment was 5.3 °C, and both motes registered temperatures differences from the recommended range of set-point ±0.5 °C, more than 98% of the time.

RH was always below the industry recommendations of 95% and sometimes as low as 55%. Significant differences were found between the RH around the product (measured by mote 3) and the RH of the ambient air above the load in the middle of the semi-trailer (mote 4), the later RF always being higher than the former.

Pressure sensors showed significant fluctuations in the operation of the cooling equipment. The recorded barometric pressure, together T and RH, were used to calculate psychrometric charts, which gave information about condensation and evaporation of water inside the transport trailer.

A complete monitoring system should include external-long range communication such us satellite or GSM (Global System for Mobile Communications) which can send real time information to a remote station. The benefit of such system is the information provided about the conditions inside the trailer all along the trip. Measurements obtained are consistent and provide valuable information on the conditions encountered during the shipment. It is possible to address, at regular time increments, what is happening with the product, whether it is temperature, humidity or acceleration.

Another advantage is not only avoiding loss of product on trucks, but also providing effective support in legal situations as well as safety inspections. Security issues are also considered, because door openings or intrusions can be detected with the light sensor.

The RF power used by the motes is not strong enough to go through the pallets, due to the high water content of the products transported. Future experiments should include the deployment of higher number of motes with more RF power (which means higher energy consumption) and RSSI recordings. Also, there is the need for testing such systems in the long-term conditions such as transoceanic shipments.

## Figures and Tables

**Table 1.** Coefficients used to compute the psychometric data [15].

R = 22,105,649.25
A = −27,405.526
B = 97.5413
C = −0.146244
D = 0.12558 × 10^−3^
E = −0.48502 × 10^−7^
F = 4.34903
G = 0.39381 × 10^−2^

**Table 2.** ANOVA of spatial T data. Fishers’s F for two factors (type of sensor and mote) and their interaction (confidence interval 95%).

	**Mote**	**Type of Sensor**	Type of Sensor*Mote
Fishers’s F	3,833.59	28.24	140.44

**Table 3.** ANOVA of RH, pressure, light and acceleration data. Fishers’s F considering node location as a factor (confidence interval 95%).

**Factor**	**RH**	**Pressure**	**Light**	**Acceleration**
Node	1,406.85	387.49	91.77	1,122.95
